# Experiential knowledge of expert coaches and expert athletes can help identify constraints on the performance of run-up in competitive sport tasks

**DOI:** 10.3389/fpsyg.2025.1544196

**Published:** 2025-02-26

**Authors:** Behrouz Ghorbanzadeh, Behzad Mohammadi Orangi, Rasoul Yaali

**Affiliations:** ^1^Department of Physical Education and Sport Sciences, Faculty of Education and Psychology, Azarbaijan Shahid Madani University Tabriz, Tabriz, Iran; ^2^Department of Sport Science, School of Humanities, Damghan University, Damghan, Iran; ^3^Faculty of Physical Education and Sport Sciences, Kharazmi University of Tehran, Tehran, Iran

**Keywords:** experiential knowledge, elite coaches and athletes, vault and long jump, penalty kick, ecological dynamics

## Abstract

**Introduction:**

Understanding athletes’ performance in competitive environments helps practitioners design practice environments to improve athletes’ skills. This study investigated the experiential knowledge of 30 elite coaches and athletes from track and field, gymnastics, and soccer, aimed at increasing understanding of individual, environmental, and task constraints on expert athletes’ performance.

**Methods:**

The interviews were conducted in a semi-structured manner and based on ecological dynamics.

**Results:**

Results show that while some constraints on run-up performance are common across all three sports, others are sport-specific. Focus, readiness, self-confidence, speed, and decision-making were identified as individual constraints. Environmental constraints included spectators, coach role, practice facilities, and competition stakes. Task constraints comprised performance order, markers, significant others in the competition, and competition timing and results.

**Discussion:**

The findings support the ecological dynamics perspective that athlete performance emerges from the interaction of environmental, task, and personal constraints. Athletes must maintain focus during run-up while managing various pressures, including crowd noise and competition stress. Coaches provide crucial technical and psychological support that enhances confidence and focus. Quality practice facilities and consistent training environments aid athletes’ spatial awareness and situational resilience. Task-specific constraints, such as performance order and timing, present unique challenges that athletes must navigate through dynamic adjustments based on real-time changes in conditions. The results contribute to the design of training environments and consequently to athletes’ performance improvement. The study suggests that coaches should design training environments that simulate real-world competitive constraints to help athletes develop adaptive skills under pressure. These findings have practical implications for designing training programs that enhance athletes’ ability to perform consistently in high-stakes competitive situations.

## 1 Introduction

In modern society, the impact of sports transcends the individual and extends to societal pride, economic contributions, and even national identity (McGarry, 2020). Competitive sports, often viewed as a reflection of a nation’s capability and resilience, have therefore garnered substantial attention not only from sports professionals but also from policymakers and economists. This attention has sparked a growing body of research dedicated to understanding the factors that contribute to optimal performance under competitive pressures, as these insights can drive more effective training approaches and refine athletic development at both amateur and professional levels (Sparkes, 2024).”

A critical yet often underexamined aspect of athletic performance is the run-up phase, which plays a decisive role in sports such as track and field, gymnastics, and soccer. Studies have shown that the quality of the run-up can significantly impact the final outcome, making it a crucial stage for athletes aiming to execute high-stakes actions like vaulting in gymnastics, long-jumping, or taking penalty kicks in soccer (Bridgett et al., 2002). During the run-up, athletes are required to harmonize physical control, mental focus, and situational awareness. Such coordination is necessary to successfully navigate various sport-specific constraints, which may include adjusting stride length, controlling speed, and managing stress induced by audience presence or the competition setting itself (Bridgett et al., 2002).

The Ecological Dynamics framework offers a powerful lens to study athletic performance as it emerges from the continuous interaction of three types of constraints: individual, environmental, and task-related (Davids et al., 2021). Individual constraints encompass physical, cognitive, and emotional factors intrinsic to the athlete, such as endurance, concentration, self-confidence, and decision-making abilities (Rothwell et al., 2023). For instance, in the context of gymnastics, an athlete’s ability to remain focused during the run-up can directly influence the control and precision needed for a successful vault.

Environmental constraints are external factors that impact an athlete’s performance, such as crowd noise, the quality of practice facilities, or the presence of coaches who may provide strategic insights and psychological support (Seifert et al., 2013). In soccer, for example, the level of crowd noise during a penalty kick can alter an athlete’s focus and increase anxiety, highlighting how situational pressures can influence the decision-making process and execution quality (Button et al., 2023).

Task constraints, on the other hand, include sport-specific demands and rules that shape how athletes approach their performance. These constraints can vary greatly between sports. In long jumping, for instance, athletes must carefully control their stride to ensure they do not cross the take-off line, while in penalty kicks, soccer players must follow a regulated distance from the ball and time their shots precisely. These task constraints thus create unique situational demands that athletes must adapt to in real time (Davids et al., 2021).”

Previous research has extensively explored the influence of constraints on athletic performance across various sports, with a predominant emphasis on coaches’ perspectives. Studies such as Greenwood et al. (2014) and McCosker et al. (2019) have highlighted how coaches’ experiential knowledge can identify key informational constraints affecting performance. However, these studies overlook the direct experiences of athletes, who are the primary agents navigating these constraints in real-time competitive settings. Similarly, Levi and Jackson (2018) examined the impact of confidence and decision-making under competitive pressure but focused solely on athletes without incorporating the strategic insights provided by coaches. Recent literature (Cassidy et al., 2024) underscores the necessity of integrating both perspectives to achieve a more comprehensive understanding of how individual, environmental, and task constraints dynamically shape performance.

Sports performance emerges from the complex interplay between coaches’ strategic guidance and athletes’ situational adaptability (Cassidy et al., 2024). While coaches contribute expertise in tactical planning, psychological conditioning, and environmental management, athletes must translate these directives into real-time execution under varying constraints (Cassidy et al., 2024). The exclusive focus on either group in previous studies has led to an incomplete depiction of the mechanisms underlying performance regulation. By simultaneously examining the perspectives of both athletes and coaches, this study bridges a critical gap in the literature, offering a holistic framework for understanding how constraints interact to influence decision-making, skill execution, and overall competitive outcomes.

This study aims to examine the impact of individual, environmental, and task constraints on the performance during the run-up phase in three competitive sports: pole vaulting, long jumping, and penalty kicks. Based on the ecological dynamics framework, this study hypothesizes that individual constraints (e.g., focus, self-confidence, decision-making), environmental constraints (e.g., spectators, coaching presence, training facilities), and task constraints (e.g., performance order, distance markers) significantly influence athletes’ run-up performance. Athletes’ responses to these constraints may be influenced by various factors, including gender. Research has shown that men and women may differ in information processing, competitive pressure management, and tactical decision-making (Jurak et al., 2023). These differences are more pronounced at competitive levels and can affect how athletes interact with different constraints (Dorfberger et al., 2009). However, including both groups in a single study may introduce methodological complexities that make result interpretation challenging (Mason, 2017). Therefore, this study intentionally focuses on male athletes to allow for a more precise analysis of the effects of different constraints on performance. This approach not only provides more coherent data but also lays the groundwork for future studies that can conduct similar investigations among female athletes and offer broader comparisons.

To gain a deeper understanding of the interaction between individual, environmental, and task constraints, this study incorporates insights from both elite coaches and athletes. This dual-perspective approach enables a more comprehensive analysis of how these constraints influence sports performance and can contribute to developing practical strategies for designing training environments that simulate competitive conditions. Such an approach allows athletes and coaches to cultivate adaptable strategies to navigate these constraints effectively (Button et al., 2023).

## 2 Methodology

### 2.1 Research approach and theoretical framework

This study employs a constructivist grounded theory approach (Puddephatt, 2006) to explore the experiences and perceptions of elite coaches and athletes regarding constraints that influence their performance during the run-up phase across three sports: track and field, gymnastics, and soccer. Constructivist grounded theory allows for a deep exploration of how participants interpret and ascribe meaning to various individual, environmental, and task constraints in competitive settings. The study is further supported by the Ecological Dynamics framework (Davids et al., 2021; Seifert et al., 2023), which is valuable for understanding the interplay of constraints in shaping athletic performance.

This framework is essential to this research because it emphasizes the adaptability of athletes to a combination of individual, environmental, and task-based factors, aligning with the study’s goal of examining how constraints dynamically influence competitive performance. The constructivist grounded theory approach enables a nuanced understanding of social and experiential meanings among participants, offering valuable insights into how athletes and coaches co-construct performance realities in real-time contexts.

### 2.2 Participants

The study selected 30 elite participants (15 coaches and 15 athletes) using purposive sampling (Patton, 2015). This technique was chosen to ensure that participants with highly specific expertise and relevant experience were included, as their insights would be particularly informative for understanding nuanced constraints in elite-level sports. The participants included coaches and athletes specializing in vaulting in gymnastics, long jump, and penalty kicks in soccer. Based on previous studies (Greenwood et al., 2014; Greenwood et al., 2012), the criteria for selecting a coach include professional coaching experience at both national team and domestic league levels, with a minimum of 10 years. The average coaching experience in this study is approximately 13 years, while athletes have an average of around 10 years of experience. Participant demographics, including experience levels and age distribution within each subgroup, are presented in Table 1.

**Table 1:** Age, number and history of participants.

participants Future		N*	Age (M-SD)*	History based on expert or elite level (year) (M-SD)
Coaches	Vault Jump	5	47.8–2.11	14.3–2.43
	Long jump	5	51.2–3.7	13.1–1.44
	Soccer penalty	5	49.7–2.9	12.4–2.52
	Total	15	49.56–2.9	13.26–2.13
Athletes	Vault Jump	5	23.6–1.77	6.77–1.23
	Long jump	5	26.7–2.41	8.31–2.4
	Soccer penalty	5	28.9–3.1	10.2–2.1
	Total	15	26.4–2.42	8.42–1.91
Total		30	37.98–2.66	10.84–2.02

*M, mean; SD, standard deviation; N, number.

The sample size estimation using GPower was solely an initial approximation to determine the preliminary number of participants, while the final sample size was completed once data saturation was achieved. This process followed the criteria proposed by Morse (2000) and Guest et al. (2006) for qualitative studies. Saturation was reached when adding new participants no longer generated novel insights, and informational patterns were sufficiently repeated. This approach ensured that the collected data possessed adequate depth and richness for analysis.

Including only male participants was an intentional methodological choice to control for potential gender-based variability in experiences and perceptions of competitive constraints. A single-gender sample can enhance the study’s validity by reducing variability caused by gender differences in competitive behavior, focus, and social interaction within sport settings. This approach is commonly employed in qualitative research when the focus is on depth rather than generalizability (Mason, 2017). By concentrating on a single-gender sample, we aimed to obtain a richer, more consistent set of insights into how male athletes and coaches navigate constraints in competitive environments, providing a foundation for future research on female athletes. This methodological choice allowed for a more focused analysis, leading to clearer patterns and more specific findings that can inform tailored coaching strategies and competitive frameworks.

### 2.3 Data collection

Data were collected through semi-structured interviews combined with video-assisted recall of successful and unsuccessful performances. This method allowed for a detailed exploration of constraints encountered in competitive performance, aligning with the Ecological Dynamics framework (Davids et al., 2015). The interview guide was carefully structured with questions categorized by individual, environmental, and task constraints. For instance, questions included “What factors do you focus on during the run-up phase?” and “How does the atmosphere of a high-stakes competition affect your performance?”

The interview questions were developed based on a thorough review of relevant literature on Ecological Dynamics framework in sports performance, ensuring alignment with established theoretical frameworks. Additionally, input from experienced coaches and sport scientists was incorporated to enhance the practical relevance of the questions. The guide was refined through multiple rounds of expert feedback, focusing on clarity, specificity, and the ability to elicit meaningful reflections from participants. This structured approach ensured that the interviews captured nuanced insights into how athletes perceive and respond to competitive constraints.

To enhance the accuracy of recall and engagement, participants were shown video footage of both successful and unsuccessful performances prior to the interview. Athletes viewed their own performances, while coaches reviewed footage of their athletes. Conducting the interviews in familiar sports facilities created a comfortable environment for the participants, and each interview lasted approximately 45–60 min. All interviews were audio-recorded and transcribed verbatim to preserve the details of participants’ insights.

### 2.4 Data analysis

Data analysis was guided by the constructivist grounded theory approach (Puddephatt, 2006), involving multiple stages of coding and constant comparison to identify and refine themes. NVivo software (Kraiwanit et al., 2023) was employed to facilitate systematic coding, enabling efficient management and analysis of complex qualitative data. Initially, open coding was conducted to generate primary codes from the transcripts, identifying preliminary themes in individual, environmental, and task constraints. These initial codes were continuously refined through an iterative constant comparison process, where codes were reviewed within and across interviews to reveal underlying patterns, relationships, and differences (Creswell and Poth, 2018). Initially, open coding was employed to identify emerging themes related to individual, environmental, and task constraints. For example, phrases such as “The athlete must block out distractions” were categorized under focus as an individual constraint, while statements like “The crowd noise made it harder to concentrate” were coded under environmental constraints. Axial coding was then used to establish relationships between categories, refining themes into broader concepts. Selective coding finalized the process by integrating key patterns into a cohesive framework that aligned with the Ecological Dynamics model.

To further validate and deepen the analysis, reflexivity practices were implemented to mitigate researcher biases, including keeping an audit trail and using reflective memoing to document personal assumptions throughout the coding and interpretation stages (Puddephatt, 2006). Regular peer debriefing sessions were held among the research team, where coded segments and emerging themes were reviewed collectively to ensure consistency of coding and interpretations. This process also involved identifying any potential biases that could inadvertently influence the thematic analysis, ensuring that participant perspectives remained the central focus (Cutcliffe and McKenna, 2004).

Additionally, member checking was conducted to enhance the credibility of the data. In this process, participants were given the opportunity to review their transcripts and confirm the accuracy of their responses, adding an extra layer of reliability to the findings.

### 2.5 Trustworthiness and rigor

To ensure the trustworthiness of the study, multiple strategies were employed. Triangulation was achieved by using both interviews and video-assisted recall, which allowed participants to anchor their responses in actual performance contexts, providing a richer perspective on their experiences. Peer debriefing sessions among the research team and member checking with participants further strengthened the credibility and dependability of the data. The use of NVivo also contributed to confirmability, as systematic data management and coding minimized potential researcher bias, thereby supporting transparent and reliable interpretations.

The study’s transferability was supported by providing detailed descriptions of the research process, sampling approach, and participant demographics, allowing readers to assess the applicability of findings to similar settings (Lincoln and Guba, 1985). The rigor of the methodology and the systematic data analysis further reinforce the dependability and validity of the study’s conclusions.

### 2.6 Ethical considerations

Ethical approval for the study was obtained from the university’s ethics committee. All participants were fully informed about the study’s objectives, procedures, and their rights, including the right to withdraw from the study at any stage without penalty. Written informed consent was obtained from all participants, ensuring confidentiality and anonymity of their data. Each participant was assigned a unique code to anonymize identities, and all audio recordings and transcripts were securely stored in compliance with ethical research guidelines (American Psychological Association, 2019).

### 2.7 Theoretical framework model

**Figure 1:**
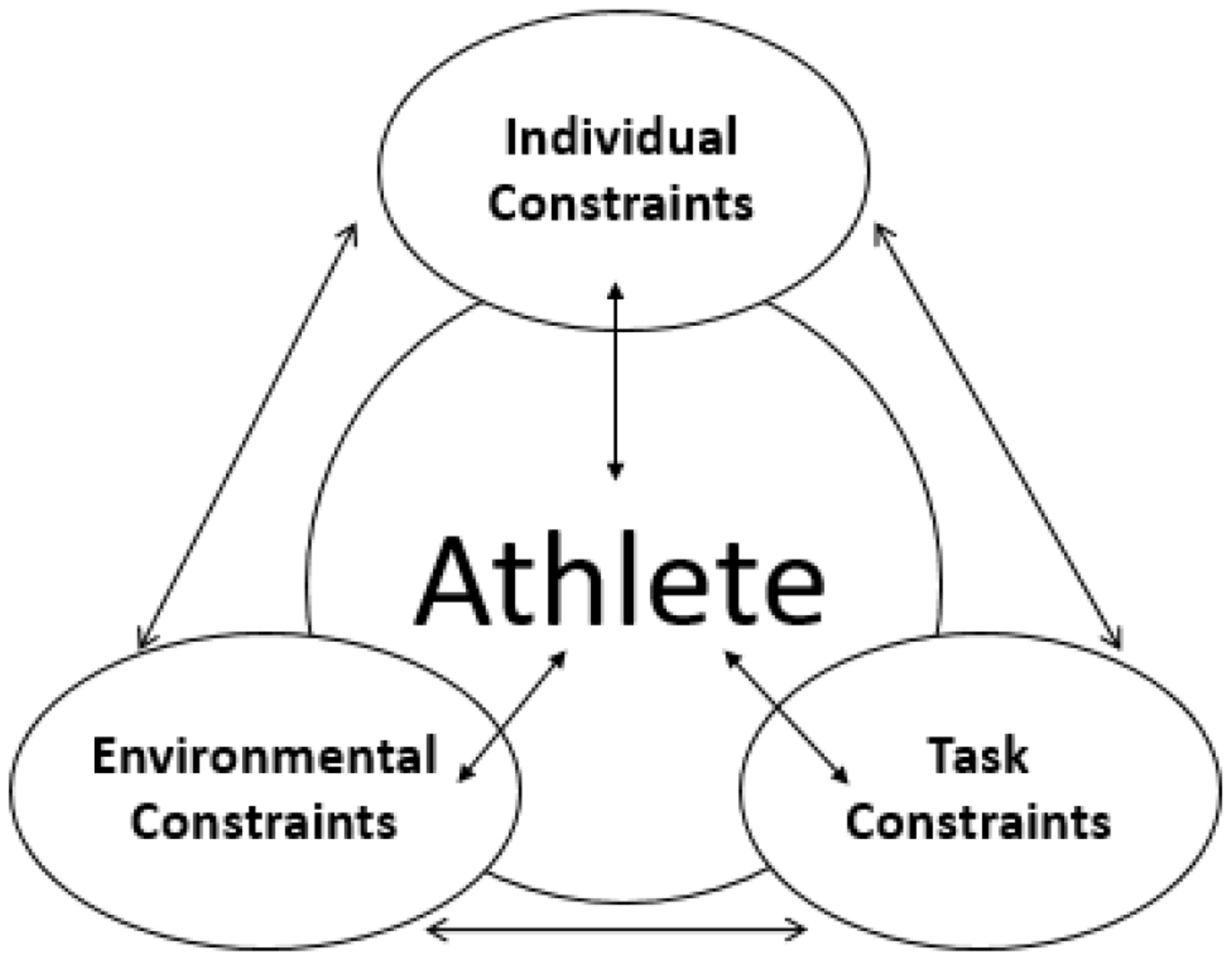
Interaction model of constraints on run-up performance.

The Ecological Dynamics model, illustrated in Figure 1, demonstrates the interaction between individual, environmental, and task constraints. In this model:

*Individual Constraints:* Represent personal attributes, such as focus, physical fitness, self-confidence, and decision-making abilities. *Environmental Constraints:* Include external factors like crowd noise, coach support, and the atmosphere of competition. *Task Constraints:* Refer to sport-specific rules, goals, and contextual factors such as distance to targets or required timings.

This model illustrates the dynamic interplay between individual, environmental, and task constraints, and how they collectively shape the athlete’s performance. The athlete is positioned at the center, where these constraints converge and influence their perceptions, decision-making, and actions. The feedback loop within the model suggests that athletes continuously adapt their behaviors in response to changes in the constraints, as they strive to optimize their performance in the competitive environment.

## 3 Results

This study examines the perceptions of elite athletes and coaches in gymnastics, track and field, and soccer regarding individual, environmental, and task constraints that impact run-up performance. These constraints affect athletes’ responses by shaping their focus, adaptability, and decision-making processes within competitive contexts. The findings are grounded in the Ecological Dynamics framework, which posits that performance emerges from interactions between these constraints. Each section concludes with a focused summary, leading to an overarching conclusion with practical applications for training and suggestions for future research. Table 2 shows the percentage of coaches and athletes who mentioned each of the constraints. Based on this table, it is clear that most coaches and athletes (over 80%) considered the constraints mentioned in this study to be important.

**Table 2:** Percentage of coaches and athletes who mentioned the identified constraints.

Constraint		Coaches’ responses (%)			Athletes’ responses (%)		
		Soccer	Vault jump	Long jump	Soccer	Vault jump	Long jump
Individual constraints	Focus	100	100	100	100	100	100
	Readiness	100	100	100	100	100	100
	Self-confidence	100	80	100	100	100	100
	Speed	100	100	100	80	100	100
	Decision-making	80	80	80	100	80	100
Environmental constraints	Spectator presence	100	80	100	100	80	80
	Coaching influence	100	80	80	80	80	100
	Practice facility quality	100	80	100	80	100	100
	Competition stakes	100	100	100	100	100	100
Task constraints	Performance order	100	100	100	100	100	100
	Markers	100	100	100	100	100	100
	Significant others	60	100	100	80	100	100
	Distance/timing	100	100	100	100	100	100

### 3.1 Individual constraints

#### 3.1.1 Focus

Athletes’ ability to selectively attend to goal-relevant information and filter out distractions emerged as a key constraint. For example, a gymnastics coach stated, “The athlete must focus on their performance while running to succeed...ignore factors that are detrimental and consider those that align with the goal” (vault jump coach, 7). In high-pressure situations, such as soccer penalty kicks, athletes must maintain focus despite crowd noise and stress. This ability to sustain attention under pressure is essential for perception-action coupling, where athletes prioritize task cues and block out disruptions.

#### 3.1.2 Readiness

Both physical and mental readiness are fundamental for elite performance, allowing athletes to meet sport-specific demands. Coaches highlighted that strength, flexibility, and mental resilience enhance an athlete’s responsiveness. As one gymnastics coach noted, “To compete effectively, the athlete must be physically and mentally prepared...fitness is essential, influencing mental readiness as well” (vault jump coach, 10). In soccer, readiness is crucial for penalty success, as both physical and mental preparation contribute to handling competitive pressure.

#### 3.1.3 Self-confidence

High self-confidence is critical, particularly in high-stakes scenarios. Confident athletes are more likely to perform optimally without hesitation. A vault jump coach observed, “The athlete must believe in themselves...confidence is key to success” (vault jump coach, 8). In soccer, confidence is especially vital during penalty shots, where athletes who doubt themselves may struggle to succeed under crowd pressure. Confidence supports perception-action coupling, enabling athletes to execute movements smoothly.

#### 3.1.4 Speed

Speed is crucial for events like long jump, where faster approaches correlate with greater jump distances. As one long jump coach stated, “In long jump, running speed is crucial...at world-class levels, speed can be a determining factor” (long jump coach, 5). Athletes also noted that speed contributes to overall performance, highlighting the need to balance speed with precision.

#### 3.1.5 Decision-making

Adaptive decision-making is essential, particularly in soccer, where athletes adjust in real-time based on the goalkeeper’s positioning. Coaches emphasized that athletes should rely on situational cues rather than rigid pre-planned responses. As one soccer coach explained, “An athlete should observe the goalkeeper and make the decision at the moment” (soccer coach, 11). This flexible approach enables athletes to respond dynamically to unfolding events, enhancing strategic effectiveness.

#### 3.1.6 Summary for individual constraints

Focus, readiness, self-confidence, speed, and decision-making collectively enhance athletes’ ability to adapt in real-time, fostering situational awareness and control. These attributes align with the Ecological Dynamics framework by preparing athletes to engage with task-relevant cues, reinforcing adaptive strategies that improve performance outcomes.

### 3.2 Environmental constraints

#### 3.2.1 Spectators

The presence of spectators can either motivate or distract athletes. In gymnastics, one coach noted, “Spectators are significant...they can motivate or disrupt focus depending on their support” (vault jump coach, 9). In soccer, opposing crowd noise can heighten stress during penalty kicks, as one athlete shared, “The opposing crowd’s cheers intensified during my penalty kick, making it hard to focus” (soccer athlete, 13). This dual influence of spectators highlights the need for mental resilience.

#### 3.2.2 Coaching influence

Coaches provide technical and psychological support that can enhance confidence and focus. In soccer, coaches reassure athletes before penalties, as one explained, “Before the penalty, I talk to the athlete...this encouragement increases their confidence” (soccer coach, 12). In gymnastics, positive reinforcement from coaches helps manage athletes’ stress and build resilience.

#### 3.2.3 Practice facilities

Quality of facilities, such as lighting and spatial arrangements, impacts athletes’ control and coordination. A gymnastics coach emphasized, “The gym lighting should be consistent so that athletes can concentrate on performance factors” (vault jump coach, 6). Consistent training environments aid athletes’ spatial awareness, reducing disorientation during competition.

#### 3.2.4 Competition stakes

High-stakes competitions introduce additional pressure, requiring athletes to maintain composure. As one soccer coach stated, “In the 90th minute, a penalty kick is highly sensitive...it can determine the game result” (soccer coach, 11). Managing high-stakes scenarios is crucial for athletes to avoid performance anxiety and execute effectively under pressure.

#### 3.2.5 Summary for environmental constraints

Spectator presence, coaching influence, practice facility quality, and competition stakes shape the external pressures that athletes must navigate. Supportive factors enhance focus and resilience, while adverse conditions demand mental toughness, supporting the Ecological Dynamics model where athletes adapt dynamically to context-driven challenges.

### 3.3 Task constraints

#### 3.3.1 Performance order

The order of performance affects athletes’ mental readiness, particularly in gymnastics and track events. A vault jump coach remarked, “Going first or following a top performer can create stress, influencing the next athlete’s performance” (vault jump coach, 7). This awareness can impact athletes’ confidence and anxiety.

#### 3.3.2 Marks and checkpoints

Distance markers in long jump serve as critical reference points for stride adjustment. As one coach explained, “Distance markers are crucial...they help athletes adjust steps approaching the jump” (long jump coach, 8). These markers improve pacing, timing, and rhythm, enhancing accuracy.

#### 3.3.3 Significant others

In soccer, players monitor the goalkeeper’s movements to make rapid, strategic adjustments. A coach explained, “Goalkeepers usually move in line...the player must anticipate and exploit these movements” (soccer coach, 11). This situational awareness during penalty kicks can increase scoring accuracy.

#### 3.3.4 Distance and timing

Managing the distance and timing of approaches impacts performance, particularly in penalty kicks where spacing affects control. Coaches advise careful management of this range to prevent telegraphing shot direction. “The distance between the player and ball must be managed carefully...to prevent compromise of shot accuracy” (soccer coach, 15).

#### 3.3.5 Summary for task constraints

Performance order, markers, significant others, and distance/timing shape athletes’ responses and enhance situational awareness, allowing for precise timing and execution. Effective adaptation to these constraints enhances consistency, underscoring the value of tactical awareness in competitive settings.

### 3.4 Importance of identified constraints for gymnastics, track and field, and soccer

Each individual constraint plays a critical role in performance across gymnastics, track and field, and soccer during the run-up phase, as reported by athletes and coaches. The significance of each constraint varies depending on the specific demands of each sport, with some constraints having a more direct impact on performance outcomes than others.

Focus is universally essential across all three sports during the run-up phase. In gymnastics, athletes must concentrate on their movements to ensure proper technique and timing during the approach to the vault. In soccer, focus is crucial for controlling the ball and evaluating the goalkeeper’s positioning during the penalty kick run-up. In track and field, athletes need to focus on their stride, timing, and rhythm to optimize their approach for takeoff in events like the long jump. Coaches emphasized that maintaining focus during this phase is critical for preventing mistakes and ensuring optimal execution. Readiness, including both physical and mental preparation, is also recognized as essential across all three sports. In soccer, athletes must be prepared both physically and mentally to handle the pressure of penalty kicks, where concentration and timing are crucial. In gymnastics, physical readiness, such as strength and flexibility, plays a vital role in performing complex movements during the run-up to the vault. Similarly, in track and field, readiness is key to optimizing approach speed, ensuring efficient strides, and preparing for takeoff in events like the long jump.

Self-confidence is vital across all sports. In soccer, confidence is particularly important during penalty kicks, where athletes need to trust in their ability to execute under pressure. In gymnastics, confidence in one’s technique is necessary to ensure successful execution during the run-up to the vault. In track and field, athletes rely on self-belief to maintain the necessary pace and control during the run-up to the long jump, where high levels of confidence enable athletes to overcome potential performance anxiety. Speed plays a significant role in track and field and gymnastics, where the speed of the run-up directly influences performance. In track and field, athletes aim to increase their approach speed to gain momentum for a stronger, longer jump. In gymnastics, a fast yet controlled approach is essential for proper timing and height during the vault. In soccer, however, the focus shifts to maintaining an optimal, controlled speed during the penalty kick approach, as excessive speed may reduce accuracy and control. Coaches emphasized that while speed is important in track and field and gymnastics, in soccer, it is crucial to balance speed with precision to avoid compromising the accuracy of the shot. Decision-making is a crucial aspect of performance across all sports, though its role varies in the run-up phase. In soccer, athletes must assess the goalkeeper’s positioning and make rapid adjustments to their strategy, often deciding how to approach the penalty kick based on the goalkeeper’s movements. In gymnastics and track and field, decision-making is vital for adjusting the stride or approach based on conditions or previous performance. For example, in gymnastics, athletes may modify their running pace to ensure proper takeoff angles, while in track and field, adjustments are made to ensure optimal jump conditions.

Coaching influence plays a significant role in all three sports. In soccer, coaches influence the mental preparation and tactical approach before the penalty kick, offering encouragement and strategic guidance to enhance performance during the run-up phase. In gymnastics, coaches provide real-time feedback, assisting athletes in refining their pacing and focus during the run-up to the vault. Similarly, in track and field, coaches work closely with athletes to optimize their approach strategy, ensuring proper timing and speed during the run-up. Spectator presence impacts performance in all three sports, though its influence varies. In soccer, the position of spectators behind the goal can increase psychological pressure during penalty kicks, with some athletes reporting that crowd noise and opposition cheers affected their focus. In gymnastics, spectators can serve as a source of motivation or distraction depending on the athlete’s ability to manage the crowd’s presence. In track and field, spectators also have a similar effect, either motivating athletes or causing distractions based on their individual mental resilience.

Task constraints, such as performance order, marks and checkpoints, significant others, and distance and timing, also affect the run-up phase in each sport. In gymnastics and track and field, the order of performance can impact athletes’ mental readiness, particularly if they perform first or after top performers. In soccer, the distance between the ball and the player during the penalty kick approach is a critical factor for control and accuracy. Coaches emphasized the importance of carefully managing the timing and distance of the run-up to ensure proper execution during the critical moments in all three sports.

These nuanced differences in the application of individual, environmental, and task constraints, as noted by athletes and coaches, demonstrate how each constraint influences performance in the run-up phase. While focus, readiness, self-confidence, speed, and decision-making are crucial in all three sports, their relative importance and specific impact on performance outcomes are shaped by the unique demands and competitive contexts of gymnastics, track and field, and soccer. The comparison highlights that while these constraints collectively shape performance, their influence is sport-specific, with some variables being more influential in particular contexts, such as the importance of speed in track and field and gymnastics compared to its role in soccer. This understanding underscores the need to consider each sport’s unique characteristics when assessing the impact of individual constraints on performance.

### 3.5 Model structure

#### 3.5.1 Individual constraints

Focus: Task-specific cues and decision-making. Readiness: Physical and mental preparation affecting response to task demands. Self-Confidence: Influences focus and ability to perform under pressure. Speed: Correlates with performance outcomes, especially in speed-dependent tasks like the long jump. Decision-Making: Impacts performance, especially in dynamic environments (e.g., soccer penalty kicks).

#### 3.5.2 Environmental constraints

Spectators: Potential to motivate or distract, affecting focus and mental resilience. Coaching Influence: Positive reinforcement, affecting confidence and emotional regulation. Practice Facilities: Quality and consistency of facilities affecting athlete coordination and comfort. Competition Stakes: Introduces pressure, influencing the ability to maintain focus and composure under high-stress conditions.

#### 3.5.3 Task constraints

Performance Order: Influence on mental readiness and confidence, especially under high-stress competition sequences. Marks and Checkpoints: Aid in pacing and rhythm, essential for performance accuracy (e.g., in the long jump). Significant Others: Tactical decisions influenced by observations of key figures like goalkeepers in soccer. Distance and Timing: Impact performance, especially in penalty kicks, where space and approach timing are critical to accuracy.

#### 3.5.4 Key feedback loop

**Figure 2:**
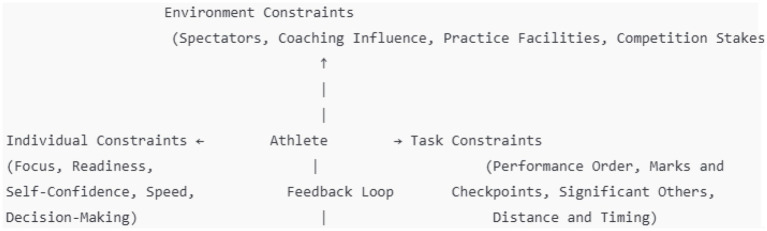
Structural model based on the results of this study: the role of individual, environment and task limitations on athlete performance.

The interaction between these constraints (individual, environmental, and task) fosters dynamic feedback loops where athletes’ responses adjust in real-time based on the ongoing integration of these constraints, enhancing performance outcomes. This feedback loop supports adaptive decision-making, situational awareness, and the development of mental resilience (Figure 2).

## 4 Discussion

This study aimed to deepen our understanding of how individual, environmental, and task constraints interact to shape elite athletes’ performance during the run-up phase in sports such as pole vault, long jump, and penalty kicks in soccer. Our findings reveal that these constraints are integral to athletes’ focus, preparedness, and adaptability. By using an Ecological Dynamics framework (Davids et al., 2021), this research highlights the continuous interactions between the individual, task, and environment as critical in determining performance outcomes in high-stakes scenarios. Importantly, this study uniquely incorporates perspectives from both athletes and coaches, offering a well-rounded view of the factors influencing athletic performance. While previous studies have often focused on coaches’ viewpoints alone (Greenwood et al., 2014; McCosker et al., 2019), the inclusion of athletes’ insights in this study provides richer data on the functional constraints directly affecting them, thus bridging a crucial research gap identified in recent literature.

One of the central findings of this research is the role of individual constraints—including focus, self-confidence, and decision-making—each of which is instrumental in navigating the competitive pressures of high-stakes sports environments. For example, based on our data, 100% of athletes and coaches acknowledged that focus is a critical factor in run-up performance across all three sports (see Table 2). This strong consensus suggests that the ability to filter distractions is universally important in performance optimization. Studies suggest that athletes’ ability to manage their attention and filter out distractions is essential for performance under competitive pressures (Bahmani et al., 2019). Decision-making, particularly in dynamic environments such as penalty kicks, is another critical skill that aligns with the ecological approach, emphasizing perception-action coupling (Button et al., 2023). Athletes must not only perceive cues (e.g., the goalkeeper’s position in soccer) but also adapt their actions based on real-time environmental input. In our study, 80% of coaches emphasized the importance of decision-making in soccer and long jump, supporting the idea that adaptability plays a significant role in successful execution. This adaptability is especially important in high-speed sports, where time constraints and pressure can challenge an athlete’s capacity to execute learned behaviors under dynamic conditions (Levi and Jackson, 2018). The insights from this study reinforce that these individual constraints are not only foundational to successful athletic performance but also critical in preparing athletes to adaptively engage with external constraints.

Environmental constraints, including crowd influence, coaching support, and training conditions, are also pivotal to athletic outcomes. Our results indicate that 80% of athletes and coaches acknowledged that spectators have a direct influence on performance, either enhancing focus or creating additional stress. Crowds can either serve as a motivator or a stressor, as previously identified by Rothwell et al. (2023), where high noise levels and intense spectator attention may heighten athletes’ anxiety, impacting performance consistency. In the current study, athletes and coaches confirmed that both supportive and adverse crowd behaviors affect their focus and emotional state, underscoring the need for resilience strategies. The influence of coaching is equally significant. Coaches provide both tactical guidance and psychological support, which positively influences athletes’ focus and confidence. For instance, 100% of coaches and 80% of athletes reported that coaching influence plays a substantial role in their preparation and execution of the run-up phase. This study aligns with Button et al. (2023), which stresses the role of coaching in enhancing athletes’ ability to manage competitive stress. Additionally, the quality of practice facilities can either enhance or inhibit performance, suggesting that controlled training environments can help athletes develop spatial awareness and situational resilience (Seifert et al., 2013). Table 2 shows that 100% of coaches in long jump and soccer highlighted practice facility quality as an important factor, reinforcing the need for high-quality training environments. These findings emphasize the necessity of designing training conditions that mirror competitive environments, allowing athletes to practice in contexts that mimic the sensory and psychological demands of competition.

Task-specific constraints, such as performance order, timing, and physical reference markers, present unique challenges across sports. Our findings reveal that 100% of long jump athletes and coaches identified markers and checkpoints as crucial to performance, as they aid in stride regulation and spatial awareness. For example, markers in the long jump provide essential visual cues that guide athletes’ pace and stride adjustments, directly impacting jump accuracy and success (Greenwood et al., 2016). The order of performance, particularly in events like gymnastics, can create psychological pressure that athletes need to manage to maintain consistency. Our study’s findings further underscore the importance of situational awareness, where athletes dynamically adjust their actions based on real-time changes in task conditions (Davids et al., 2021). Data from this study indicate that 80% of soccer athletes highlighted the importance of monitoring the goalkeeper’s movements during the run-up phase, further confirming that decision-making within task constraints is a crucial factor. These adaptations are central to the Ecological Dynamics perspective, where task constraints continuously shape athletes’ decisions and responses. The findings suggest that task constraints should be systematically integrated into training to cultivate adaptive behaviors and enhance task-specific skills across diverse sport settings.

### 4.1 Practical implications for training design

From a practical perspective, this research underscores the value of training environments that replicate real-world constraints, enabling athletes to cultivate adaptive skills under simulated competitive pressures. By incorporating crowd noise, varied task orders, and other situational constraints, trainers and coaches can foster environments where athletes are more resilient and adaptable (Davids et al., 2021). For example, in sports such as soccer, coaches might incorporate penalty kick practices that simulate actual game conditions, with varying crowd noise and time pressure to improve athletes’ adaptability and focus. This aligns with Seifert et al. (2013), which encourages a constraints-led approach to training that reflects the nuanced interactions between an athlete’s internal and external demands. The athlete responses in this study emphasize that training under realistic conditions, including varied environmental and psychological stressors, improves focus, confidence, and overall performance. The incorporation of situational constraints within training environments not only builds physical and mental preparedness but also improves athletes’ confidence in handling unpredictable competition dynamics, thereby supporting higher performance consistency.

### 4.2 Study limitations and future directions

This study makes a novel contribution by integrating both athlete and coach perspectives within the Ecological Dynamics framework. Unlike previous research that predominantly focused on either group separately, this dual-perspective approach offers a more holistic understanding of how constraints interact in competitive settings. By considering both the experiential knowledge of coaches and the real-time adaptations of athletes, these findings emphasize the importance of designing training environments that closely replicate competition conditions, ensuring athletes develop the necessary adaptive skills for high-performance scenarios.

While this study provides robust insights, it is limited to male athletes from three specific sports, potentially constraining the generalizability of the findings. Although efforts were made to ensure a diverse and representative sample within these constraints, the limited number of participants may not fully capture the variability of experiences across different levels of competition and athletic backgrounds. Future research could examine female athletes across additional sports to explore potential gender-based differences in responses to similar constraints, especially given that competitive pressures and coping mechanisms may vary between genders.

Furthermore, while this study identifies key individual, environmental, and task constraints, it may not encompass all possible factors influencing performance. Elements such as psychological conditions, social dynamics, and injury history could also play significant roles but were beyond the primary scope of this investigation. Although qualitative methods provide in-depth insights into these constraints, they do not quantify findings or establish statistical significance, which may limit the extent to which conclusions can be broadly applied to all athletes and settings. However, the use of a rigorous qualitative approach, including theoretical saturation, systematic coding, and researcher triangulation, enhances the reliability of the findings. Future studies incorporating mixed-methods approaches or larger, more diverse samples could further validate and expand upon these insights, ensuring a more comprehensive understanding of the complex interactions shaping athletic performance.

## 5 Conclusion

In conclusion, this study highlights that elite athletic performance during the run-up phase in competitive sports is shaped by a dynamic interplay of individual, environmental, and task constraints. Supported by the Ecological Dynamics framework, our findings suggest that training environments should emulate real-world constraints to foster athletes’ adaptive capacities and improve their competitive performance. By understanding and integrating these constraints into training designs, coaches and practitioners can better support athletes in achieving resilience and adaptability, essential qualities for success in high-performance sports.

The insights gained from this study are not limited to the three sports examined—track and field, gymnastics, and soccer—but can be applied to a wider range of sports that involve preparatory movement phases under competitive conditions. Many sports, such as basketball, tennis, and swimming, require athletes to manage similar constraints, including maintaining focus, adjusting movement strategies, and responding to environmental and situational pressures. By tailoring training programs to incorporate sport-specific constraints while maintaining the core principles identified in this study, coaches can enhance athletes’ ability to perform consistently across different disciplines. Additionally, the findings are relevant to a broader athlete population, as the interaction between individual, environmental, and task constraints is fundamental to skill acquisition and performance optimization across varying levels of expertise and competitive settings.

## Data Availability

The raw data supporting the conclusions of this article will be made available by the authors, without undue reservation.
